# Evaluation of post-vaccination immunoglobulin G antibodies and T-cell immune response after inoculation with different types and doses of SARS-CoV-2 vaccines: A retrospective cohort study

**DOI:** 10.3389/fmed.2022.1092646

**Published:** 2023-01-10

**Authors:** Rami H. Al-Rifai, Farida Alhosani, Rowan Abuyadek, Shereen Atef, James G. Donnelly, Andrea Leinberger-Jabari, Luai A. Ahmed, Basel Altrabulsi, Adnan Alatoom, Ahmed R. Alsuwaidi, Laila AbdelWareth

**Affiliations:** ^1^Institute of Public Health, College of Medicine and Health Sciences, United Arab Emirates University, Al Ain, United Arab Emirates; ^2^Zayed Center for Health Sciences, United Arab Emirates University, Al Ain, United Arab Emirates; ^3^Abu Dhabi Public Health Center–ADPHC, Abu Dhabi, United Arab Emirates; ^4^High Institute of Public Health, Alexandria University, Alexandria, Egypt; ^5^National Reference Laboratory, Abu Dhabi, United Arab Emirates; ^6^Faculty of Medicine, Ain Shams University, Cairo, Egypt; ^7^Public Health Research Center, New York University, Abu Dhabi, United Arab Emirates; ^8^Pathology and Laboratory Medicine Institute (PLMI), Cleveland Clinic Abu Dhabi, Abu Dhabi, United Arab Emirates; ^9^Department of Pediatrics, College of Medicine and Health Sciences, United Arab Emirates University, Al Ain, United Arab Emirates

**Keywords:** SARS-CoV-2, COVID-19, vaccination, coronavirus, vaccine

## Abstract

**Introduction:**

The induction and speed of production of severe acute respiratory syndrome coronavirus-2 (SARS-CoV-2) immune biomarkers may vary by type and number of inoculated vaccine doses. This study aimed to explore variations in SARS-CoV-2 anti-spike (anti-S), anti-nucleocapsid (anti-N), and neutralizing immunoglobulin G (IgG) antibodies, and T-cell response by type and number of SARS-CoV-2 vaccine doses received.

**Methods:**

In a naturally exposed and SARS-CoV-2–vaccinated population, we quantified the anti-S, anti-N, and neutralizing IgG antibody concentration and assessed T-cell response. Data on socio-demographics, medical history, and history of SARS-CoV-2 infection and vaccination were collected. Furthermore, nasal swabs were collected to test for SARS-CoV-2 infection. Confounder-adjusted association between having equal or more than a median concentration of the three IgG antibodies and T-cell response by number and type of the inoculated vaccines was quantified.

**Results:**

We surveyed 952 male participants with a mean age of 35.5 years ± 8.4 standard deviations. Of them, 52.6% were overweight/obese, and 11.7% had at least one chronic comorbidity. Of the participants, 1.4, 0.9, 20.2, 75.2, and 2.2% were never vaccinated, primed with only one dose, primed with two doses, boosted with only one dose, and boosted with two doses, respectively. All were polymerase chain reaction-negative to SARS-CoV-2. BBIBP-CorV (Sinopharm) was the most commonly used vaccine (92.1%), followed by rAd26-S + rAd5-S (Sputnik V Gam-COVID-Vac) (1.5%) and BNT162b2 (Pfizer-BioNTech) (0.3%). Seropositivity to anti-S, anti-N, and neutralizing IgG antibodies was detected in 99.7, 99.9, and 99.3% of the study participants, respectively. The T-cell response was detected in 38.2% of 925 study participants. Every additional vaccine dose was significantly associated with increased odds of having ≥median concentration of anti-S [adjusted odds ratio (aOR), 1.34; 95% confidence interval (CI): 1.02–1.76], anti-N (aOR, 1.35; 95% CI: 1.03–1.75), neutralizing IgG antibodies (aOR, 1.29; 95% CI: 1.00–1.66), and a T-cell response (aOR, 1.48; 95% CI: 1.12–1.95). Compared with boosting with only one dose, boosting with two doses was significantly associated with increased odds of having ≥median concentration of anti-S (aOR, 13.8; 95% CI: 1.78–106.5), neutralizing IgG antibodies (aOR, 13.2; 95% CI: 1.71–101.9), and T-cell response (aOR, 7.22; 95% CI: 1.99–26.5) although not with anti-N (aOR, 0.41; 95% CI: 0.16–1.08). Compared with priming and subsequently boosting with BBIBP-CorV, all participants who were primed with BBIBP-CorV and subsequently boosted with BNT162b2 had ≥median concentration of anti-S and neutralizing IgG antibodies and 14.6-time increased odds of having a T-cell response (aOR, 14.63; 95% CI: 1.78–120.5). Compared with priming with two doses, boosting with the third dose was not associated, whereas boosting with two doses was significantly associated with having ≥median concentration of anti-S (aOR, 14.20; 95% CI: 1.85–109.4), neutralizing IgG (aOR, 13.6; 95% CI: 1.77–104.3), and T-cell response (aOR, 7.62; 95% CI: 2.09–27.8).

**Conclusion:**

Achieving and maintaining a high blood concentration of protective immune biomarkers that predict vaccine effectiveness is very critical to limit transmission and contain outbreaks. In this study, boosting with only one dose or with only BBIBP-CorV after priming with BBIBP-CorV was insufficient, whereas boosting with two doses, particularly boosting with the mRNA-based vaccine, was shown to be associated with having a high concentration of anti-S, anti-N, and neutralizing IgG antibodies and producing an efficient T-cell response.

## 1. Introduction

Since the early 20th century, vaccines have proven to be effective tools for controlling and eliminating life-threatening infectious diseases. On 3 December 2022, more than 649.67 million cases and 6.64 million deaths have been reported, as the coronavirus disease 2019 (COVID-19) pandemic continues (1). In 185 countries, approximately 19.8 million lives were saved in the first year of COVID-19 vaccination. This estimate corresponds to a 63% reduction in COVID-19-related deaths in the absence of vaccines (2). Humoral and cellular immune responses are the main drivers of protection against severe acute respiratory syndrome coronavirus-2 (SARS-CoV-2). While neutralizing antibodies (Nabs) established a clear role in protection against infection, particularly in the early post-vaccination period (3, 4), cellular immunity is also proven to alleviate the disease severity and enhance recovery (5). The currently approved and available vaccines have different mechanisms of action in triggering the immune system to produce immune response biomarkers that predict vaccine effectiveness. Several studies have discussed the vaccine effect of various vaccine types on inducing immune responses to produce immune biomarkers and their durability (4–8). However, these studies were limited in examining the effect of whole inactivated vaccine, the effect of vaccine mixing between more than one type, and the change in immunity levels by the number and type of the received vaccine doses on different forms of immunity.

In the United Arab Emirates (UAE), which hosts the world’s most fully vaccinated population (9), five types of vaccines were approved for emergency use to control the spread of the SARS-CoV-2 virus. These approved vaccines are BBIBP-CorV (commercial name: Covilo, Sinopharm’s Beijing Institute of Biological Products), BNT162b2 (commercial name: Comirnaty, Pfizer-BioNTech), rAd26-S + rAd5-S (commercial name: Sputnik V, Gamaleya Research Institute of Epidemiology and Microbiology), ChAdOx1-S (commercial name: Vaxzevria, AstraZeneca-University of Oxford), and mRNA-1273 (commercial name: Spikevax, Moderna-NIAID) (10). Understanding the impact of different vaccine types and the number of vaccine doses in enhancing the immune response will help inform policymakers on future vaccination and immunization strategies. Therefore, this retrospective cohort study aimed to investigate the variation in the immune response to SARS-CoV-2 using the concentration of the anti-spike (anti-S), anti-nucleocapsid (anti-N), and neutralizing immunoglobulin G (IgG) antibodies and T-cell response among a cohort of participants who were previously seropositive to SARS-CoV-2 before the emergency use of vaccines in the Emirate of Abu Dhabi, UAE.

## 2. Materials and methods

### 2.1. Study population

The study included male participants who were naturally exposed to SARS-CoV-2 (tested seropositive to anti-S IgG and anti-N IgG antibodies) in a previously published cross-sectional study that covered 24 workstations for participants across the Emirate of Abu Dhabi. Following the random sampling approach, the cross-sectional study surveyed 4,855 male expatriate workers residing in 40 workstations across the Emirate of Abu Dhabi. Of the 3,585 seropositive workers, 952 workers were available between 3 October 2021 and 15 December 2021. The available workers were surveyed and retrospectively followed in this study. More details about that study are available elsewhere (11). Participants enrolled in the cross-sectional study were invited back and asked to re-consent to participate in the current study.

### 2.2. Survey data collection, nasal swab, and blood sampling

Participants available at the time of the survey were invited to participate in this study and provide blood samples. They filled in an online survey that collected data on their socio-demographics, existing chronic medical conditions, smoking status, body weight and height, and a history of testing positive for SARS-CoV-2 infection. Two whole blood samples (5–7 ml each) were collected from each participant in plain tubes and in a tube with an anticoagulant. Moreover, at the survey time, a nasopharyngeal swab was collected from each participant for SARS-CoV-2 testing using reverse transcription-polymerase chain reaction (RT-PCR). Collected blood samples in plain tubes were preserved at suitable conditions for serum separation and screening for three humoral SARS-CoV-2 IgG immune biomarkers [anti-spike (anti-S), anti-nucleocapsid (anti-N), and neutralizing IgG antibodies]. The other collected whole blood samples were screened for T-cell response. Malaffi (“my file” in Arabic), an Abu Dhabi-based central medical record database (12), was used to retrieve data on the history of vaccination against SARS-CoV-2, including the number and type of the vaccine doses received and the date of each dose, and the history of SARS-CoV-2 RT-PCR testing before blood collection. Any vaccination that occurred on or after the study of blood sampling was not counted.

### 2.3. Laboratory work: Immune biomarker testing

#### 2.3.1. NAb immunoassays

Testing the collected blood samples for NAbs against the SARS-CoV-2 receptor-binding domain (RBD) was performed using the iFlash-2019-nCoV NAb kit, a one-step competitive chemiluminescence immunoassay (CLIA) on the iFlash 1800 analyzer (YHLO Biotech Co., Ltd., Shenzhen, China) according to the manufacturer’s instructions.

#### 2.3.2. Anti-S and anti-N IgG immunoassays

Two types of SARS-CoV-2 IgG antibodies were measured. The first assays released at the start of the pandemic were used. The first assay to measure the anti-N IgG antibodies was the SARS-CoV-2 nucleocapsid total antibodies, which we analyzed using the Roche Cobas 6000 platform (Roche Diagnostics International AG, Rotkreuz, Switzerland). This assay was CE marked/FDA EUA-approved. The second assay that quantified the anti-S IgG antibodies was the SARS-CoV-2 Trimeric S IgG using the DiaSorin LIAISON^®^ (Saluggia, Italy) SARS-CoV-2 Trimeric S IgG. This assay is an indirect CLIA technology for the detection of IgG antibodies to SARS-CoV-2 Spikes 1 and 2 (S1 and S2) and RBD and is calibrated against the World Health Organization standard.

#### 2.3.3. T-cell response

The interferon-gamma release assay (IGRA) (QuantiFERON™, Qiagen) was utilized as a marker for T-cell activation. The Qiagen QuantiFERON SARS-CoV-2 (QFN SARS-CoV-2) blood collection kit consists of two antigen tubes (long and short peptides), SARS-CoV-2 Ag1 and SARS-CoV-2 Ag2, which uses a combination of antigens specific to SARS-CoV-2 to stimulate lymphocytes in heparinized whole blood involved in cell-mediated immunity. Plasma from the stimulated samples was used for the detection of interferon-gamma (IFN-γ) using QuantiFERON ELISA. There is a null control to baseline circulating IFN-γ and a positive T-cell control tube using mitogen as the lymphocyte stimulant.

Table 1 presents the sensitivity, specificity, and lowest detection limit for the used laboratory assays.

**TABLE 1:** Sensitivity, specificity, and the lowest detection limit for the used laboratory assays.

Assay type	Sensitivity	Specificity	Lowest detection limit
LIAISON^®^ SARS-CoV-2 TrimericS IgG	Days post-positive PCR ≥15 (94.5–99.6%)	99.7%	4.81 BAU/ml
Elecsys^®^ Anti-SARS-CoV-2	Days post-positive PCR ≥14 days (97.0–100%)	99.80%	Qualitative test COI < 1.0–non-reactive COI ≥ 1.0 Reactive
iFlash-2019-nCoV Neutralization Antibody Test	95.4%–post-vaccination 90%–post-infection	98.0%	4 AU/ml
QuantiFERON SARS-CoV-2 (The QFN SARS-CoV-2 BCTs are for Research Use Only)	80.12%	92.99%	Qualitative test

#### 2.3.4. SARS-CoV-2 RT-PCR testing

Viral RNA amplification was performed using the NeoPlex™ COVID-19 detection kit. RT-PCR was used for the RNA detection targeting N gene, *ORF1a* PCRC (SolGent Co., Ltd. Daejeon, Korea).

### 2.4. Statistical analyses

Socio-demographics and other characteristics were described using frequency distributions and measures of central tendency. For continuous measures, means and standard deviations (SDs) were reported. The distribution of the measured three humoral immune biomarkers (anti-S, anti-N, and neutralizing IgG antibodies) was described using medians and the interquartile ranges. The normality assumption for the distribution values of the IgG antibodies was investigated using the Shapiro–Wilk test. Even after implementing several transforming strategies and investigating the normality assumption of residuals, the IgG antibody values violated the normality assumption. To be used as a binary outcome variable, each of the three measured IgG antibody biomarkers was subsequently categorized using the median value into two categories (<median or ≥median). Based on T-cell response, the participants were categorized into with responding or with nonresponding T-cell. Following the manufacturer’s instructions, the responding T-cell was defined if the Ag1 or Ag2 antigen minus Nil (≤8.0 IU/ml) were ≥0.15 and ≥25% of Nil while the non-responding T-cell was defined if the Ag1 and Ag2 antigen minus Nil (≤8.0 IU/ml) were <0.15 or ≥0.15 and <25% of Nil and the Mitogen minus Nil is ≥0.50. The correlation between the level of each of the three measured SARS-CoV-2 IgG antibodies sero-biomarkers (<median vs. ≥median) and T-cell response (responding or non-responding) by the measured characteristics and history of vaccination was investigated. Chi-squared or Fisher’s exact tests were used for categorical characteristics, and the two-sample non-parametric Mann–Whitney U-test was used for continuous characteristics. The distribution of, stratified by the type and number of vaccine doses received, anti-S, anti-N, and neutralizing IgG antibody concentration was plotted and presented in boxplots. The *P*-value for this comparison was elicited from the non-parametric independent samples of the Kruskal–Wallis test. The distribution of participants with T-cell response by type and number of vaccine doses received was plotted and presented in stacked bar graphs.

Univariate and multivariable binary logistic regression models were used to estimate the crude (OR) and adjusted odds ratio (aOR). The multivariable regression model was adjusted for age, body mass index (BMI), smoking, number of vaccine doses, type of vaccine (except for only-BBIBP-CorV-vaccinated), history of the previous SARS-CoV-2 infection, and time duration in days since the last vaccine dose received and blood collection. All vaccines received on the same date of or after blood collection were not considered. All statistical analyses were performed using SPSS IBM Statistics software (v26). *P*-values of <0.05 were considered statistically significant.

## 3. Results

### 3.1. Baseline characteristics

The enrolled 952 male participants were retrospectively followed up from the last vaccine dose received until blood collection for a mean time of 89.2 days ± 54.5 SD. The participants had a mean age of 35.5 years (±8.4 SD). The majority (92.5%) were of Asian nationalities, and 52.7% were with primary education or below. Of this population, 21.4% were current smokers, and 52.6% were overweight (40.9%) or obese (11.7%). Approximately, 11.0% of the participants reported having at least one chronic comorbidity. The most common comorbidities were high blood pressure (7.0%) followed by diabetes mellitus (4.1%) (Table 2). None of the participants reported having any immunodeficiency conditions or taking any immunosuppressive medications.

**TABLE 2:** Distribution of the study population by their measured socio-demographic and clinical characteristics and their correlation with the four-tested immune response biomarkers.

	*N* = 952 (valid %)	Anti-S IgG *N* = 952 (*n*, valid %)		*P*-value	Anti-N IgG *N* = 952 (*n*, valid %)		*P*-value	Neutralizing IgG *N* = 952 (*n*, valid %)		*P*-value	T-cell response *N* = 925 (*n*, valid %)		*P*-value
		**Mean: 648.1 ± 641.7 SD Median = 357.5 stockticker BAU/ml** **(IQR: 173–930.5)** **Range: 25–2,080**			**Mean: 145.0 ± 74.8 SD Median = 146.5 COI** **(IQR: 92.0–205.5)** **Range: 0.0–320.0**			**Mean: 363.1 ± 339.4 SD Median = 172.0 AU/ml** **(IQR: 51–800)** **Range: 3–810**			**Yes** **353 (38.2%)**	**No** **572 (61.8%)**	
		** <357.5**	** ≥357.5**		** <146.5**	** ≥146.5**		** <172.0**	** ≥172.0**				
Age median, IQR–year (range, mean ± SD)	35.0, 29.0–41 (20–65, 35.5 ± 8.40)	34.0, 28.0–41.0 (34.7 ± 8.4)	36.0, 30.0–42.0 (36.3 ± 8.4)	0.003^1^	34.0, 28.0–41.0 (35.1 ± 8.5)	36.0, 30.0–42.0 (36.0 ± 8.3)	0.049^1^	34.0, 28.0–41.0 (34.8 ± 8.3)	36.0, 30.0–42.0 (36.2 ± 8.5)	0.018^1^	36.0, 30.0–42.0 (36.6 ± 8.7)	36.0, 29.5–42.0 (34.8 ± 8.2)	0.002^1^
*Missing*	*5*												
Nationality				<0.001			0.360			<0.001			0.017
Asian	881 (92.5)	424 (48.1)	457 (51.9)		441 (50.1)	440 (49.9)		423 (48.0)	458 (52.0)		335 (39.1)	523 (60.9)	
African	66 (6.9)	50 (75.8)	16 (21.2)		31 (47.0)	35 (53.0)		51 (77.3)	15 (22.7)		15 (23.8)	48 (76.2)	
Others	5 (0.5)	2 (40.0)	3 (60.0)		4 (80.0)	1 (20.0)		3 (60.0)	2 (40.0)		3 (75.0)	1 (25.0)	
Education													
Primary education and below^2^	502 (52.7)	–	–	–	–	–	–	–	–	–	–	–	–
Secondary education	352 (37.0)	–	–	–	–	–	–	–	–	–	–	–	–
University and postgraduate levels	93 (9.8)	–	–	–	–	–	–	–	–	–	–	–	–
*Missing*	*5*												
Tobacco smoking				0.030			0.507			0.258			0.780
Current smoker	203 (21.4)	116 (57.1)	87 (42.9)		106 (52.2)	97 (47.8)		112 (55.2)	91 (44.8)		76 (39.0)	119 (61.0)	
Ex-smoker	45 (4.7)	26 (57.8)	19 (42.2)		25 (55.6)	20 (44.4)		22 (48.9)	23 (51.1)		15 (33.3)	30 (66.7)	
Never-smoker	699 (73.4)	332 (47.5)	367 (52.5)		341 (48.8)	358 (51.2)		340 (48.6)	359 (51.4)		260 (38.1)	422 (61.9)	
*Missing*	*5*										*2*	1	
Received flu shot				0.082			0.559			0.082			0.027
Yes	3 (0.3)	0 (0.00)	3 (100)		2 (66.7)	1 (33.3)		0 (0.0)	3 (100)		348 (37.9)	571 (62.1)	
No	944 (99.2)	474 (50.2)	470 (49.8)		470 (49.8)	474 (50.2)		474 (50.2)	470 (49.8)		3 (100)	0 (0.0)	
*Missing*	*5*										*2*	*1*	
BMI, median, IQR (mean: 25.3 ± 3.8 SD)	25.2, 22.6–27.7	25.0, 22.3–27.7	25.4, 22.8–27.6	0.117	25.3, 22.6–27.9	25.2, 22.6–27.3	0.528	25.2, 22.6–27.8	25.2, 22.6–27.4	0.875	25.4 22.8–28.0	25.07 22.5–27.5	0.050^1^
Underweight	28 (3.2)	15 (53.6)	13 (46.4)	0.339	13 (46.4)	15 (53.6)	0.972	18 (64.3)	10 (35.7)	0.149	8 (28.6)	20 (71.4)	0.576
Normal weight	386 (44.2)	200 (51.8)	186 (48.2)		197 (51.0)	189 (49.0)		186 (48.2)	200 (51.8)		140 (37.3)	235 (62.7)	
Overweight	357 (40.9)	173 (48.5)	184 (51.5)		180 (50.4)	177 (49.6)		183 (51.3)	174 (48.7)		136 (39.0)	213 (61.0)	
Obese	102 (11.7)	43 (42.2)	59 (57.8)		52 (51.0)	50 (49.0)		43 (42.2)	59 (57.8)		41 (42.3)	56 (57.7)	
*Missing*	*5*										*2*	*1*	
BMI, median, IQR (mean: 25.3 ± 3.8 SD)	25.2, 22.6–27.7	25.0, 22.3–27.7	25.4, 22.8–27.6	0.117	25.3, 22.6–27.9	25.2, 22.6–27.3	0.528	25.2, 22.6–27.8	25.2, 22.6–27.4	0.875	25.4 22.8–28.0	25.07 22.5–27.5	0.050^1^
Underweight	28 (3.2)	15 (53.6)	13 (46.4)	0.339	13 (46.4)	15 (53.6)	0.972	18 (64.3)	10 (35.7)	0.149	8 (28.6)	20 (71.4)	0.576
Normal weight	386 (44.2)	200 (51.8)	186 (48.2)		197 (51.0)	189 (49.0)		186 (48.2)	200 (51.8)		140 (37.3)	235 (62.7)	
Overweight	357 (40.9)	173 (48.5)	184 (51.5)		180 (50.4)	177 (49.6)		183 (51.3)	174 (48.7)		136 (39.0)	213 (61.0)	
Obese	102 (11.7)	43 (42.2)	59 (57.8)		52 (51.0)	50 (49.0)		43 (42.2)	59 (57.8)		41 (42.3)	56 (57.7)	
*Missing*	*79*										*28*	*48*	
Chronic comorbidities				0.178			0.025			0.343			0.069
No	843 (89.3)	427 (50.7)	416 (49.3)		410 (48.6)	433 (51.4)		426 (50.5)	417 (49.5)		304 (37.1)	516 (62.9)	
Yes, at least one^3^	101 (10.7)	44 (43.6)	57 (56.4)		61 (60.4)	40 (39.6)		46 (45.5)	55 (54.5)		46 (46.5)	53 (53.5)	
*Missing*	*8*										*3*	*3*	
Tested PCR positive in the past 12 months				<0.001			0.004			<0.001			0.518
No	830 (87.8)	453 (54.6)	377 (45.4)		429 (51.7)	401 (48.3)		451 (54.3)	379 (45.7)		307 (37.9)	503 (62.1)	
Yes	115 (12.2)	21 (18.3)	94 (81.7)		43 (37.4)	72 (62.6)		22 (19.1)	93 (80.9)		46 (41.1)	66 (58.9)	
*Missing*	*7*											*3*	

^1^P-values extracted from the non-parametric Mann–Whitney U-test comparing distribution across groups.

^2^99 with no education.

^3^39 (4.1%), 66 (7.0%), 17 (1.8%), 2 (0.2%), 2 (0.2%), and 1 (0.1) with diabetes mellitus, high blood pressure, hyperlipidemia, heart problem, asthma/COPD disease, and cancer, respectively.

### 3.2. SARS-CoV-2 vaccination status

Before blood collection, the majority of the 952 workers were fully vaccinated and boosted with one vaccine dose (75.2%) or primed with two vaccine doses (20.2%). Only 2.2% were fully vaccinated and boosted with two additional vaccine doses. Few workers were never vaccinated or only partially vaccinated at the time of blood collection (1.4 and 0.9%, respectively). Overall, 77.5% of the participants were boosted with at least one dose. Regardless of the number of vaccine doses, 92.2, 1.5, 0.3, 4.0, and 0.7% of the participants were vaccinated with BBIBP-CorV only, rAd26-S + rAd5-S, BNT162b2 only, primed with BBIBP-CorV and boosted with BNT162b2, and had mixed vaccine types, respectively. The mean duration since the last received vaccine dose and blood collection for immune biomarker measurement was 89.2 days (±54.5 SD). Only 2.0% were vaccinated within the past 14 days, 3.6% were vaccinated within 15–30 days, and the majority (92.8%) were vaccinated for more than 30 days before blood collection (Table 3).

**TABLE 3:** Distribution of the study population by their vaccination status and history of testing PCR-positive and their correlation with the four-tested immune response biomarkers.

	*N* = 952 (valid%)	Anti-S IgG *N* = 952		*P*-value	Anti-N IgG *N* = 952		*P*-value	Neutralizing IgG *N* = 952		*P*-value	T-cell response *N* = 925		*P*-value
		**<357.5**	**≥357.5**		**<146.5**	**≥146.5**		**<172.0**	**≥172.0**		**Yes** **353 (38.2%)**	**No** **572 (61.8%)**	
Vaccination against SARS-CoV-2				<0.001			<0.001			<0.001			0.001
Not vaccinated	13 (1.4)	9 (69.2)	4 (30.8)		9 (69.2)	4 (30.8)		9 (69.2)	4 (30.8)		4 (30.8)	9 (69.2)	
Only one dose	9 (0.9)	4 (44.4)	5 (55.6)		3 (33.3)	6 (66.7)		5 (55.6)	4 (44.4)		4 (44.4)	5 (55.6)	
Two doses	192 (20.2)	96 (50.0)	96 (50.0)		126 (65.6)	66 (34.4)		89 (46.4)	103 (53.6)		66 (35.5)	120 (64.5)	
One booster dose (three doses)	714 (75.2)	366 (51.3)	348 (48.7)		326 (45.7)	388 (54.3)		371 (52.0)	343 (48.0)		263 (37.8)	433 (62.1)	
Two booster dose (four doses)	21 (2.2)	1 (4.8)	20 (95.2)		12 (57.1)	9 (42.9)		1 (4.8)	20 (95.2)		16 (84.2)	3 (15.8)	
*Missing*	*3*											*2*	
Boosted vs. not boosted (*n* = 927)^1^				*1.00*			<*0.001*			*0.296*			*0.384*
Not boosted (two doses only) (mean duration: 159.6 ± 71.8 days)^2^	192 (20.7)	96 (50.0)	96 (50.0)		126 (65.6)	66 (34.4)		89 (46.4)	103 (53.6)		66 (35.5)	120 (64.5)	
Boosted (mean duration: 70.2 ± 24.8 days)^2^	735 (79.3)	367 (49.9)	368 (50.1)		338 (46.0)	397 (54.0)		372 (50.6)	363 (49.4)		279 (39.0)	436 (61.1)	
Vaccine type				<0.001			<0.001			<0.001			<0.001
BBIBP-CorV only (mean duration: 87.5 51.0 days)^2^	874 (92.2)	461 (52.7)	413 (47.3)		420 (48.1)	454 (51.9)		459 (52.5)	415 (47.5)		309 (36.4)	540 (63.6)	
rAd26-S + rAd5-S only (mean duration: 210 ± 13.0 days)^2^	14 (1.5)	0 (0.0)	14 (100)		14 (100)	0 (0.0)		0 (0.0)	14 (100)		5 (35.7)	9 (64.3)	
BNT162b2 only (mean duration: 106.7 ± 12.5 days)^2^	3 (0.3)	0 (0.0)	3 (100)		3 (100)	0 (0.0)		0 (0.0)	3 (100)		3 (100)	0 (0.0)	
Started BBIBP-CorV boosted with BNT162b2 (mean duration: 70.2 ± 69.2 days)^2^	38 (4.0)	5 (13.2)	33 (86.8)		24 (63.2)	14 (36.8)		5 (13.2)	33 (86.8)		27 (73.0)	10 (27.0)	
Mixed vaccine type^3^ (mean duration: 154.7 ± 64.4 days)^2^	7 (0.7)	1 (14.3)	6 (85.7)		6 (85.7)	1 (14.3)		2 (28.6)	5 (71.4)		5 (71.4)	2 (28.6)	
Not vaccinated (8) or the first dose was after blood collection (5)	13 (1.4)	9 (69.2)	4 (30.8)		9 (69.2)	4 (30.8)		9 (69.2)	4 (30.8)		4 (30.8)	9 (69.2)	
*Missing*	*3*											*2*	
Duration since last vaccine dose to blood collection, median (IQR), mean ± (SD)^4^	79.0 (56.0–96.0) 89.2 (±54.5) days			0.492		<0.001				0.873			<0.001
1–14 days	19 (2.0)	11 (57.9)	8 (42.1)		16 (84.2)	3 (15.8)		8 (42.1)	11 (57.9)		14 (77.8)	4 (22.2)	
15–30 days	34 (3.6)	14 (41.2)	20 (58.8)		16 (47.1)	18 (52.9)		16 (47.1)	18 (52.9)		16 (48.5)	17 (51.1)	
31–60 days	256 (27.0)	122 (47.7)	134 (52.3)		93 (36.3)	163 (63.7)		126 (49.2)	130 (50.8)		69 (28.0)	177 (72.0)	
61–295 days	624 (65.8)	319 (51.1)	305 (48.9)		339 (54.3)	285 (45.7)		315 (50.5)	309 (49.5)		248 (40.6)	363 (59.4)	
T-cell response (*n* = 925)				<0.001			0.975			<0.001			
Yes	353 (38.2%)	144 (40.7)	209 (59.3)		178 (50.3)	175 (49.7)		138 (39.0)	215 (61.0)		–	–	
No	572 (61.8%)	321 (56.1)	251 (43.9)		287 (50.2)	285 (49.8)		326 (57.0)	246 (43.0)		–	–	

^1^Excluding not vaccinated or received only one dose.

^2^Mean time duration post-last vaccine dose.

^3^Started with rAd26-S + rAd5-S and boosted with BNT162b2 or BBIBP-CorV (n = 5) or first dose was BBIBP-CorV and second dose BNT162b2 or rAd26-S + rAd5-S (n = 2).

^4^Included only received at least one vaccine dose.

### 3.3. Anti-S, anti-N, and neutralizing IgG antibody concentration

Seropositivity to anti-S, anti-N, and neutralizing IgG antibodies was detected in 99.7% (seropositive ≥33.8 BAU/ml), 99.9% (seropositive ≥1 COI), and 99.3% (seropositive ≥10 AU/ml) of the participants, respectively. The mean (±SD) and median concentrations of the anti-S, anti-N, and neutralizing IgG antibodies were 648.1 BAU/ml (±641.7) and ≥357.5 BAU/ml, 145.0 COI (±74.8) and ≥146.5 COI, and 363.1 AU/ml (±339.4) and ≥172.0 AU/ml, respectively. Participants who had ≥median concentration of any of the three IgG antibodies had significantly higher mean age (all *p* < 0.05). Of the participants who had ≥median concentration of anti-S, anti-N, and neutralizing IgG antibodies, 77.1, 75.2, and 75.4% never smoked tobacco, respectively. Of the participants who had at least one chronic comorbidity, 56.4, 39.6, and 54.5% had ≥median concentration of anti-S, anti-N, and neutralizing IgG antibodies, respectively (Table 2).

**FIGURE 1:**
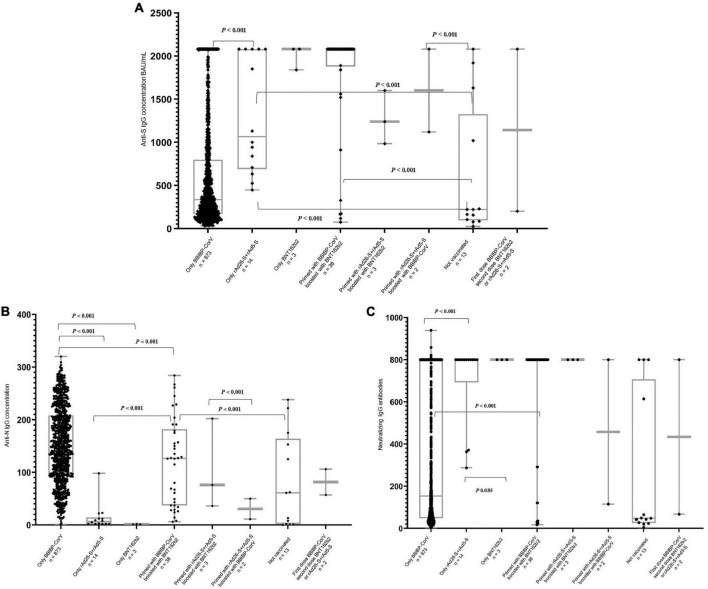
Distribution of the anti-S **(A)**, anti-N **(B)**, and neutralizing **(C)** IgG antibodies concentration by type of the received SARS-CoV-2 vaccines regardless of the total number of doses. *P*-values extracted from the Independent-Samples Kruskal–Wallis Test.

Most of the participants who had ≥median concentration of anti-S (≥357.5 BAU/ml), anti-N (≥146.5 COI), and neutralizing IgG antibodies (≥172.0 AU/ml) were boosted with at least one booster dose (79.3, 85.6, and 77.7%, respectively). Regardless of the number of the received vaccine doses, 47.3, 51.9, and 47.5% of the participants who were vaccinated with only the BBIBP-CorV vaccine had ≥median concentration of anti-S, anti-N, and neutralizing IgG antibodies, respectively. All of the 14 participants who were vaccinated with rAd26-S + rAd5-S only or BNT162b2 only (*n* = 3) had ≥median concentration of anti-S and neutralizing IgG antibodies although <median concentration of anti-N IgG antibodies (Table 3 and Figures 1A–C).

### 3.4. T-cell response

**FIGURE 2:**
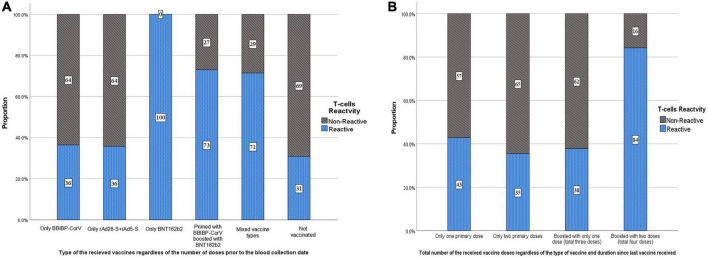
Distribution of the study participants by T-cells reactivity (response) according to the **(A)** type and **(B)** total number of the received SARS-CoV-2 vaccine doses.

Of the 925 participants, 38.2% had a T-cell response. Participants with responding T-cells had a significantly higher mean age than those with non-responding T-cells (36.6 vs. 34.8 years, *p* = 0.002) (Table 2). Of the participants who were boosted with only one dose or with two doses, 38.0 and 84.2% had a T-cell response, respectively. Overall, of the 353 participants who had a T-cell response, 79.0% were boosted with at least one dose. Of the only-BBIBP-CorV-vaccinated participants, 36.4% had a T-cell response. Of the only rAd26-S + rAd5-S-vaccinated participants, 35.7% had a T-cell response. All of the three BNT162b2-vaccinated participants had responding T-cells. Of the participants who had a T-cell response, 59.3, 49.7, and 61.0% had ≥median concentration of anti-S, anti-N, and neutralizing IgG antibodies (Table 3 and Figures 2A, B).

### 3.5. History of SARS-CoV-2 infection and immune response

Compared with other participants, a statistically significantly higher proportion of individuals having ≥median concentration of anti-S (81.7% vs. 45.4%), anti-N (62.6% vs. 48.3%), and neutralizing IgG antibodies (80.9% vs. 45.7%) was noted in participants with a history of contracting COVID-19 during the past 12 months of blood collection (Table 2). In addition, participants with a history of SARS-CoV-2 infection had a significantly (*p* < 0.001) higher mean IgG antibody concentration than those with no infection in the past 12 months (Table 4). The T-cell response was not statistically significantly associated with a history of SARS-CoV-2 infection during the past 12 months (*p* = 0.518) (Table 5).

**TABLE 4:** Crude (OR) and adjusted odds ratio (aOR) of the association between every dose increase in number of anti-SARS-CoV-2 vaccine doses and boosting status with having ≥median concentration of anti-S IgG (≥357.5 BAU/ml), anti-N IgG (≥146.5 COI), and neutralizing IgG (≥172.0 AU/ml) antibodies and having a T-cell response.

	Anti-S IgG Abs (≥median vs. <median concentration)		Anti-N IgG Abs (≥median vs. <median concentration)		Neutralizing IgG Abs (≥median vs. <median concentration)		T-cell response (Yes vs. No)	
	**OR** **(95% CI)**	**aOR** **(95% CI)**	**OR** **(95% CI)**	**aOR** **(95% CI)**	**OR** **(95% CI)**	**aOR** **(95% CI)**	**OR** **(95% CI)**	**aOR** **(95% CI)**
Additional one vaccine dose	1.22 (0.98–1.53)	1.34 (1.02–1.76)*	1.55 (1.22–1.97)***	1.35 (1.03–1.75)*	1.15 (0.92–1.44)	1.29 (1.00–1.66)*	1.26 (0.99–1.60)	1.48 (1.12–1.95)**
Booster status^1^								
Not boosted (primed with only two doses)	1.00	1.00	1.00	1.00	1.00	1.00	1.00	1.00
Boosted once (received three doses)	0.95 (0.69–130)	0.90 (0.65–1.25)	2.28 (1.64–2.18)***	2.17 (1.54–3.1)***	0.80 (0.59–1.10)	0.78 (0.56–1.09)	1.10 (0.80–1.55)	1.07 (0.76–1.51)
Boosted twice (received four doses)	20.0 (2.63–152.0)**	14.20 (1.85–109.4)*	1.43 (0.57–3.57)	1.27 (0.48–3.36)	17.30 (2.27–131.36)***	13.60 (1.77–104.3)*	9.70 (2.272–34.50)***	7.62 (2.09–27.87)**
Vaccine type–only vaccinated^2^								
BBIBP-CorV only	1.00	1.00	1.00	1.00	1.00	1.00	1.00	1.00
Primed with BBIBP-CorV boosted with BNT162b2	7.39 (2.86–19.09)***	7.57 (2.61–21.94)***	0.54 (0.27–1.06)	0.48 (0.23–1.0)	7.32 (2.83–18.9)***	7.86 (2.71–22.83)***	4.72 (2.25–9.90)***	4.28 (1.93–9.50)***
rAd26-S + rAd5-S only	NA	NA	NA	NA	NA	NA	0.97 (0.32–2.92)	1.13 (0.36–3.60)
Others^3^	10.01 (1.27–79.8)*	9.95 (1.23–80.65)*	0.10 (0.01–0.81)*	0.14 (0.02–1.1)	4.41 (0.94–21.0)	4.14 (0.85–20.14)	7.00 (1.48–33.12)*	7.90 (1.63–38.6)*
Others^4^	25.74 (3.46–191.41)**	29.52 (3.9–223.9)***	0.04 (0.005–0.30)**	0.06 (0.01–0.48)**	25.73 (3.50–191.41)***	13.52 (3.06–59.2)***	2.07 (0.91–4.70)	2.46 (1.03–5.9)*

Adjusted odds ratio for age (continuous), BMI (continuous), type of vaccine, smoking status, chronic comorbidity, time duration since last vaccine dose, and history of previous infection (PCR+).

^1^Adjusted also for type of vaccine.

^2^Adjusted also for total number of vaccine doses.

^3^Three of them received only BNT162b2, and the rest received heterogeneous vaccine types.

^4^Three of them received only BNT162b2, and 14 received only rAd26-S + rAd5-S. The rest received heterogeneous vaccine types.

***P < 0.001, **P = 0.002, and *P < 0.005.

**TABLE 5:** Correlation between history of SARS-CoV-2 infection during the past 12 months and anti-S, anti-N, and neutralizing IgG antibody concentration and T-cell response during follow-up.

	Anti-S IgG Abs BAU/ml		Anti-N IgG Abs COI		Neutralizing IgG Abs AU/ml		T-cell response		*P*-value
**Contracted COVID-19 in the past 12 months**	**Mean ± SD**	**Mean difference** ** *P*-value**	**Mean difference** ** *P*-value**		**Mean difference** ** *P*-value**		**Yes**	**No**	
No	594.4 ± 623.9	438.3, *P* < 0.001	144.8 ± 75.2	25.3, *P* < 0.001	330.4 ± 332.4	266.2, *P* < 0.001	37.9%	62.1%	0.518
Yes	1,032.7 ± 647.6		170.1 ± 68.01		596.3 ± 296.7		41.1%	58.9%	

### 3.6. Having ≥median concentration of anti-S, anti-N, and neutralizing IgG antibodies by number and type of received vaccine doses at baseline

An increase in the number of received vaccine doses by one dose was significantly associated with increased odds of having, at the survey time, ≥median concentration of the anti-S [aOR, 1.34; 95% confidence interval (CI): 1.02–1.76], anti-N (aOR, 1.35; 95% CI: 1.03–1.75), and neutralizing IgG antibodies (aOR, 1.29; 95% CI: 1.00–1.66) and a T-cell response (aOR, 1.48; 95% CI: 1.12–1.95) (Table 4). Compared with participants who were primed with two doses, those boosted with only one more dose had significantly similar (*P* > 0.05) odds, whereas those who were boosted with two more doses had increased odds of having ≥median concentration of anti-S (aOR, 14.2; 95% CI: 1.85–109.4), neutralizing IgG (aOR, 13.6; 95% CI: 1.77–104.3), and T-cell response (aOR, 7.6; 95% CI: 2.09–27.8) (Table 4).

Within boosted with at least one dose, compared with boosting with only one dose, boosting with two doses was significantly associated with increased odds of having ≥median concentration of anti-S (aOR, 13.8; 95% CI: 1.78–106.5), neutralizing IgG antibodies (aOR, 13.2; 95% CI: 1.71–101.9), and T-cell response (aOR, 7.22; 95% CI: 1.99–26.5) although not with anti-N IgG antibody (aOR, 0.41; 95% CI: 0.16–1.08). Compared with priming and boosting with BBIBP-CorV, all of the 29 participants who were primed with BBIBP-CorV and boosted with BNT162b2 had ≥median concentration of anti-S and neutralizing IgG antibodies and 14.6-time increased odds of having a T-cell response (aOR, 14.63; 95% CI: 1.78–120.5). Every additional dose of the BBIBP-CorV vaccine was not significantly (*P* > 0.05) associated with any observed increased odds of having ≥median concentration of the measured three immunoglobulin types or with T-cell response. A similar finding was observed when comparing BBIBP-CorV boosted with BBIBP-CorV-non-boosted participants (Table 6).

**TABLE 6:** Crude (OR) and adjusted odds ratio (aOR) of the association between every dose increase in the number of anti-SARS-CoV-2 vaccine doses and boosting status with having ≥median concentration of anti-S IgG (≥357.5 BAU/ml), anti-N IgG (≥146.5 COI), and neutralizing IgG (≥172.0 AU/ml) antibodies and having a T-cell response.

	Anti-S IgG Abs (≥median vs. <median concentration)		Anti-N IgG Abs (≥median vs. <median concentration)		Neutralizing IgG Abs (≥median vs. <median concentration)		T-cell response (Yes vs. No)	
	**OR** **(95% CI)**	**aOR** **(95% CI)**	**OR** **(95% CI)**	**aOR** **(95% CI)**	**OR** **(95% CI)**	**aOR** **(95% CI)**	**OR** **(95% CI)**	**aOR** **(95% CI)**
Booster status								
Boosted once (received three doses)	1.00	1.00	1.00	1.00	1.00	1.00	1.00	1.00
Boosted twice (received four doses)	21.1 (2.82–158.0)**	13.8 (1.78–106.54)*	0.53 (0.26–1.51)	0.41 (0.16–1.08)	21.70 (2.90–162.53)**	13.18 (1.71–101.9)*	8.78 (2.53–30.42)***	7.22 (1.99–26.25)**
Vaccine type–only boosted^1^								
Primed and boosted with BBIBP-CorV (*n* = 704)	1.00	1.00	1.00	1.00	1.00	1.00	1.00	1.00
Primed with BBIBP-CorV boosted with BNT162b2	All the 29 were with ≥median concentration		0.50 (0.23–1.08)	–	All the 29 were with ≥median concentration		10.14 (3.48–29.56)***	14.63 (1.78–120.47)*
Others^3^	–	–	–		–	–		
Only-BBIBP-CorV–vaccinated								
Additional one vaccine dose	1.09 (0.80–1.55)	0.87 (0.57–1.33)	1.57 (1.15–2.16)**	1.03 (0.68–1.55)	0.97 (0.72–1.33)	0.75 (0.49–1.13)	1.14 (0.82–1.56)	1.24 (0.79–1.94)
Booster status								
Not boosted (primed with only two doses)	1.00	1.00	1.00	1.00	1.00	1.00	1.00	1.00
Boosted once (received three doses)	1.18 (0.94–1.67)	0.99 (0.61–1.59)	1.93 (1.36–2.73)***	1.24 (0.77–1.97)	0.97 (0.69–1.37)	0.71 (0.44–1.14)	1.23 (0.85–1.78)	1.35 (0.82–2.24)
Boosted twice (received four doses)	Only one case							

Adjusted odds ratio for age (continuous), BMI (continuous), type of vaccine (except for only-BBIBP-CorV–vaccinated), smoking status, chronic comorbidity, the time duration since the last vaccine dose, and history of previous infection (PCR+).

^1^Adjusted also for the total number of vaccine doses.

^3^Only one individual primed with rAd26-S + rAd5-S and boosted with one BBIBP-CorV dose.

***P < 0.001, **P = 0.002, and *P < 0.005.

## 4. Discussion

In a population with a history of natural exposure to SARS-CoV-2 before vaccination, we investigated the association between the number and type of inoculated SARS-CoV-2 vaccine doses and the concentration of the induced immune biomarkers (anti-S, anti-N, and neutralizing IgG antibodies and T-cell response). The antibody response was tested and detected in all participants; however, T-cell response was detected in only 38.2% of the participants. Having above the median concentration of the three measured IgG antibodies and the T-cell response was associated with being primed or boosted with mRNA-based vaccines and with inoculation with two but not one booster dose. The T-cell response was significantly associated with having above the median concentration of anti-S and neutralizing IgG antibodies. Furthermore, the T-cell response was significantly associated with increased odds of being boosted with two doses although not being boosted with only one dose of the SARS-CoV-2 vaccine. Among only-BBIBP-CorV–vaccinated participants, no difference in the measured four biomarkers was observed between boosted with only one BBIBP-CorV dose and with two BBIBP-CorV doses.

A significant mean age-related difference in all the studied immune biomarkers was observed. The study participants with a mean age of 36 years had a higher concentration (≥median) of IgG and NAbs as well as responding T-cells. It was unexpected that increasing age would be associated with greater SARS-CoV-2 IgG antibodies (13). However, this cohort only included individuals in the middle-aged working group (mean age: 35.5 ± 8.4 years). It has been reported that middle-aged adults in general have the most significant immune response (14). No significant difference in the concentration of the measured IgG antibodies or T-cell response by the BMI status of this population was noted. Although obesity has been established as a risk factor for mortality from COVID-19 (15), there is no established evidence of the association between the immune response to natural infection or vaccination and BMI. Never-smoking participants had a significantly higher anti-S IgG antibody concentration than those who currently smoke. The insufficient immune response among smokers is consistent with the established evidence that current smokers had an increased risk of severe COVID-19 disease (16).

In this study, participants who tested PCR-positive in the past 12 months had a higher concentration of the measured three IgG antibodies although a lower proportion of participants had detectable T-cell response than those with a negative PCR test. During the first few months, after SARS-CoV-2 infection, the T-cell response typically wanes at a slower rate than IgG antibodies (17–20). In this study, the Qiagen IGRA was utilized to study T-cell response. This assay measures IFNg release from activated T-cells upon stimulation and is a very general measure of T-cell function and may have lower sensitivity than other methods of assessing T-cell functions. Other studies assessing T-cell functions in vaccinated populations demonstrated approximately 100% of individuals have detectable responding T-cells 6 months after at least two vaccine doses; therefore, the rate of 38% in seropositive individuals at baseline and approximately all vaccinated individuals (79% with three doses) is remarkably low. Some of this may be related to the type of vaccine administered as most of our study population received the BBIBP-CorV vaccine. Typically, the presence of T-cells and antibodies is associated with the successful resolution of average cases of SARS-CoV-2; however, high heterogeneity has been observed in studies of adaptive immunity in patients with a variable magnitude of antibody responses to SARS-CoV-2, as well as in the magnitude of SARS-CoV-2–specific CD4+ and CD8+ T-cell responses (21, 22). We still have limited data on the correlation between antibody responses to natural infection or vaccination and T-cell responses measured using the QFN SARS assay. Nevertheless, data originating from one study suggest that in individuals vaccinated with mRNA vaccines, both humoral and cellular responses are detectable using the SARS-CoV-2 serological assay and the QFN SARS assay; however, the extent of correlation is inconclusive (23).

The post-vaccination SARS-CoV-2–induced immune response varied by the number and type of vaccine doses received. An increase in the number of vaccine doses by one dose was associated with increased odds of having more than the median concentration of the IgG antibodies and with producing responding T-cells, particularly among those who were boosted with two doses. This increased immune potency by increasing the number of vaccine doses supports the significance of boosting susceptible populations to avoid exposure and re-exposure, thereby expediting the process of pandemic containment.

Regarding the association between the type of the SARS-CoV-2 vaccine and the immune status, the results showed that the levels of the measured immune biomarkers varied according to the different types of studied vaccines, even after controlling for potential confounders, including the number of vaccine doses received, exposure to SARS-CoV-2 in the past 12 months, the time since the last exposure, age, and commodities. Populations who received only or boosted with an inactivated whole virus-based vaccine (BBIBP-CorV) were less likely to have a high anti-S or neutralizing IgG antibody concentration than those who received only or boosted with at least one dose of an mRNA-based (BNT162b2) or adenovirus-based (rAd26-S + rAd5-S) vaccine. This is also consistent with the findings of other studies, wherein a significant boost of anti-S IgG antibody after the second dose of the BNT162b2 vaccine was observed (13, 24). A previous study reported that the BNT162b2 vaccine is associated with producing a high peak of anti-S IgG responses (13). In fact, expediting the time in achieving high anti-S and neutralizing IgG antibody concentrations would play a significant role in protecting individuals and saving lives amid highly transmissible pandemics. This observed expedited immune response following mRNA-based vaccination compared with other vaccine types explains the reported high effectiveness of such vaccine types in preventing infection or disease progression in different population groups (25–28). Nonetheless, in the initial stages of the pandemic when no vaccines were available, there is no doubt that the emergency authorization and use of non-mRNA-based vaccines played a significant role in reducing the risk of viral transmission and alleviating the burden of the pandemic.

Although T-cells are generated following vaccination, they usually contract from the peak within 3 months (29). In this study, more participants with a T-cell response were within the first 2 weeks after their last vaccine dose, and significantly more participants who had no T-cell response were more than 1 month after their last vaccine dose. This seems to contradict what has been previously reported in studies where T-cell responses were better 6 months following vaccination (30), and T-cell responses decline at a slower rate than the antibody levels (31). However, investigating the variation in T-cell response according to the number and type of vaccine doses, populations boosted with one or two doses or those who received an inactivated whole virus vaccine type (BBIBP-CorV) and subsequently boosted with BNT162b2 vaccine were more likely to maintain T-cell response than their counterparts. It was previously reported that the IFNg-secreting SARS-CoV-2–specific T-cells were associated with a milder form of COVID-19 disease (32). SARS-CoV-2–specific T-cells are elicited during acute COVID-19, and T-cell memory pools durability sustain for up to 8 months. Following vaccination, a 10-fold increase in IFNg-secreting T-cells was observed. Data on SARS-CoV-2–specific CD4+ and CD8+ T-cells clearly demonstrate the generation of long-term immunological epitope-specific memory T-cell pools following mRNA COVID-19 vaccination (5). During vaccination with mRNA-based vaccine-induced T-cells, after the first vaccination, with peak responses after the second immunization, memory CD4+ T-cells were detectable in 85–100% of mRNA-based vaccine recipients at 6 months after immunization (31).

This study had some limitations. First, studying only previously naturally exposed middle-aged male participants without having a comparison group of never-naturally exposed and female gender would limit the generalizability and external validity of the present findings to the wider population. Second, the lack of baseline information prior to vaccination and during the follow-up on the concentration of the measured immune response biomarkers may limit the observed difference between the number/type of the received vaccines and the status of the measured immune biomarkers. Lastly, the small number of participants within a specific vaccine type or the number of vaccine dose groups also imposed a limitation on the present findings. Nevertheless, despite these limitations and the limitations of the retrospective nature of the study design (potential effect of unmeasured or uncontrolled confounding), the present study was unique in terms of studying several types of commonly authorized and used SARS-CoV-2 vaccines as well as the number of vaccine doses received by our studied population. Moreover, this study was unique in terms of investigating the post-vaccination immune response of three humoral immune biomarkers in the same population in addition to the T-cell response. Several studies have investigated the association between only one or two SARS-CoV-2 vaccines with only one or two immune response biomarkers (13, 33, 34).

## 5. Conclusion

Inducing humoral and T-cell response varies with the type and number of vaccine doses received as well as with mixing different types of vaccine platforms. To induce a high immune response and expedite achieving a high concentration of humoral immunity that plays a significant role in neutralizing viral particles, boosting a population’s immunity with at least one booster dose is critical. Priming or boosting with mRNA-based vaccines was more potent for inducing high levels of humoral and T-cell response compared with other vaccine types. Present findings can inform policymakers and the public health system in designing future vaccination campaigns and allocating vaccination resources in the best way to achieve the accepted immune levels and protect populations.

## Data availability statement

The raw data supporting the conclusions of this article will be made available by the corresponding author. However, restrictions apply to the availability of the data. Data will be made available upon reasonable request and with permission of the ethical approval provider.

## Ethics statement

This study was reviewed and approved by the UAE National COVID-19 Research Ethics Committee (reference number: DOH/CVDC/2021/856 and amendment number: DOH/CVDC/2021/1703). From each participant, consent to collect survey information, blood sample, and nasopharyngeal swab, was obtained. The participants provided their written informed consent to participate in this study.

## Author contributions

RHA, SA, and LAW designed and conceived the study. RHA and LAA analyzed and interpreted the data. RA wrote the manuscript, and all co-authors provided input. All authors contributed to questionnaire development, and data collection, extracted the data from medical records, and coded the data, read, and approved the final manuscript.
